# Associations between individual and structural level racism and gestational age at birth in the Nulliparous Pregnancy Outcomes Study: Monitoring mothers-to-be

**DOI:** 10.21203/rs.3.rs-3898223/v1

**Published:** 2024-01-30

**Authors:** Veronica Barcelona, LinQin Chen, Yihong Zhao, Goleen Samari, Catherine Monk, Rebecca McNeil, Andrea A Baccarelli, Ronald Wapner

## Abstract

The purpose of this study was to investigate the associations between multilevel racism and gestational age at birth among nulliparous non-Hispanic Black, non-Hispanic White and Hispanic women. We conducted a secondary analysis of data of the nuMoM2b Study (2010-2013) to examine the associations between individual and structural-level experiences of racism and discrimination and gestational age at birth among nulliparous women (n=7,732) at eight sites across the U.S. Measures included the individual Experiences of Discrimination (EOD) scale and the Index of Concentration (ICE) at the Extremes to measure structural racism. After adjustment,we observed a significant individual and structural racism interaction on gestational length (p=0.03). In subgroup analyses, we found that among these with high EOD scores, women who were from households concentrated in the more privileged group had significantly longer gestations (β = 1.07, 95% CI: 0.24, 1.90). Women who reported higher EOD scores and more economic privilege had longer gestations, demonstrating the moderating effect of ICE as a measure of structural racism. In conclusion, ICE may represent a modifiable factor in the prevention of adverse birth outcomes in nulliparas.

## Introduction

Maternal stress, including experiences of racism, has been hypothesized as a root cause of perinatal health inequities in the United States (U.S.).[1] Racism is a pervasive issue that can manifest in various forms such as individual bias, institutional practices, and societal norms, and it has serious and lasting impacts on individuals' health and well-being.[2,3] Individual, or personally-mediated racism, is defined as differential assumptions about people and actions towards them based on race, while structural racism is defined as differential access to goods, services, and opportunities in society based on race.[4] Studies have found that a high proportion of Black and Hispanic pregnant women, ranging from 55–80%, report experiencing racism, with the highest occurrence of racism being at work, getting medical care, or at school.[5–8] Additional research is necessary to better understand the relationship between individual experiences of discrimination and structural racism and the PTB outcomes among Hispanic women as well. Hispanic ethnicity represents a heterogenous group of people from Latin America and is distinct from racial group identification, which may be Black, White, Mixed or Mestizo, or other.[9]

Previous studies have separately examined the association between individual exposure to racial discrimination and adverse birth outcomes, and the relationship between structural racism and adverse birth outcomes,[1,8,10] but no study has comprehensively investigated the interaction of individual experiences of discrimination and structural-level racism within the same cohort. There is also little research examining individual and structural racism among Hispanic women, and their association with gestational age. The purpose of this study was to examine the associations between individual and structural-level experiences of racism and discrimination and gestational age at birth among non-Hispanic (NH) Black, NH White, and Hispanic nulliparous women from the Nulliparous Pregnancy Outcomes Study: Monitoring mothers-to-be (nuMoM2b) study, a large, longitudinal study funded by the

National Institutes of Health.[11] Our hypothesis was that NH Black and Hispanic women with higher individual Experiences of Discrimination (EOD) scores and more disadvantage in one measure of structural racism would have shorter gestational length than White women.

### Methods

We conducted a secondary analysis of data from the nuMoM2b study, a multicenter prospective cohort study of nulliparous pregnant women from 2010–2013. Study procedures have been previously described.[12] Women with singleton pregnancies between 6–13 weeks were recruited (N = 10,038) from eight academic medical centers to examine maternal, placental, and fetal development risk factors and adverse pregnancy outcomes. Data collection included participant interview, self-administered questionnaires, chart abstraction, clinical, and biological measures. Exclusion criteria for nuMoM2b included maternal age < 13 years of age, fetal malformation, pregnancy lasting < 20 weeks' gestation, and planned termination.

## Exposures

## Individual experiences of discrimination

The Experiences of Discrimination (EOD) scale measures self-reported individual experiences of racism and discrimination in adults of all races and ethnicities from working class backgrounds, with high reliability (Cronbach's alpha = .74) in diverse samples.[5] Respondents indicate (yes/no) if the situation has happened to them and provide the reason for the perceived discrimination. Participants in nuMoM2b completed these questions asking about discrimination related to race, ethnicity, and skin color during their second trimester study visit (between 16 + 0 and 21 + 6 weeks gestation). Although the EOD is a subjective measure, it is strongly associated with psychological distress, stress biomarkers, and disease outcomes.[13–15] Total responses can be examined both continuously and categorized as low (none of the situations), medium (1 or 2), or high (3 + of the situations),[5] or a dichotomous variable defined as low (0–2 of the situations) vs. high (3 + of the situations) levels.[16] In this study, we used the dichotomous version of the EOD measure.

## Structural racism measure

We used the income and racial polarization index (ICE) [17,18] as our measure of structural racism. This was calculated: *Income = (A-P) / T*, where A = number of NH White households with income >$100,000 or higher (privileged group); P = number of NH Black households with income <$25,000 (deprived group); T = total number of NH Black and NH White households by census tract. The range for this index is from − 1 (all households in deprived group) to 1 (all households in the privileged group).

As household income data are not part of the 2010 decennial census, we used household counts from the 2010 Census[19] and the relevant household income counts from the 2014 American Community

Survey.[20] ICE was calculated using these combined data sources. This introduced a temporal discontinuity in the counts and so there are a small number of tracts for which ICE was outside of the standard bounds of the metric. We winsorized those data to the closest bounded extreme (negative extreme (-1) and positive extreme (+ 1)) in order to retain the ICE measures that are outliers.[21]

Race and ethnicity. We limited analyses to participants with complete data on our exposures and outcomes who self-identified as NH Black, NH White, and Hispanic (n = 7,732). Participants who identified as Asian or "other" in nuMoM2b were too few to be included in the current analyses. Less than one percent of participants in our sample identified with both a racial and ethnic group (i.e., Hispanic Black), therefore, we analyzed groups separately. Race and ethnicity were assessed at the baseline interview. We conceptualize race and ethnicity in this study as sociopolitical variables, not as biological variables.

## Outcome

Gestational age. Gestational age at birth was calculated as per the nuMoM2b study protocol and used a combination of last menstrual period, ultrasound, and chart abstraction to determine the most accurate due date and was measured in weeks. Participants were excluded from the present analyses if their births occurred before 22 weeks gestation, as this is often used as the minimum age of fetal viability.[22] As the participants were primiparas, we did not expect large differences in preterm birth rates as a binary variable, thus, we focused on differences in continuous gestational age.

## Covariates

We controlled for a number of covariates available in the nuMoM2b data related to individual-level differences in maternal characteristics. These include maternal age, education, smoking status, gestational hypertension, preeclampsia, and gestational diabetes.

Maternal age. This was treated as a continuous variable and calculated by subtracting the date of baseline interview from self-reported date of birth.

Education. Participants indicated the number of years of completed education at the baseline interview.

Smoking. Participants were coded as ever-smokers (yes/no) if they smoked tobacco during 3 months prior to pregnancy at the baseline interview.

Descriptive statistics were calculated for individual predictor variables and the ICE structural racism measure to describe maternal sociodemographic characteristics. Our primary aim was to assess whether there was a significant individual by structural racism effect on the gestational age at birth both in unadjusted and adjusted models controlling for maternal age, education, smoking, gestational hypertension, preeclampsia, and gestational diabetes. Linear-mixed effects models were used, where study site was treated as a random effect to account for variability from different sites (between subjects). Our main findings remained significant when EOD was treated as a three-level (low, medium, and high) categorical variable. All analyses were performed in R, and we received Institutional Board Approval from Columbia University for the current analysis (AAAU0215).

## Results

Among the 7,732 participants included in this analysis, 67.7% identified as NH White (n = 5,234), 13.9% NH Black (n = 1,073), and 18.4% Hispanic (n = 1,425). More than half of participants (55.6%) were between age 20 and 29, and a majority had at least a high school education (Table 1). On average, women gave birth at 38.9 weeks gestation and 8.3% (n = 640) of women had preterm births less than 37 weeks. Approximately 18% smoked tobacco in the three months prior to pregnancy, and women reported an average of 0.4 lifetime racial discrimination experiences (SD = 1.0; range = 0 - 9), and 91% of participants reported less than three experiences of discrimination on the EOD measure. The mean value for ICE measures was 0.1 (SD = 0.3), and 24% were in households concentrated in the deprived group (ICE < 0).

Among Hispanic women (n = 1,425), the majority (86.1%) were interviewed in English (data not shown). Approximately a third (38.6%) were first (born in the U.S.) or second generation (women and their parents were born in the U.S.) (36.1%). The most frequently represented country of origin among foreign-born participants was the Dominican Republic (43.5%), followed by Mexico (27.5%) and U.S. citizens from the territory of Puerto Rico (5.1%). Almost all Hispanic women in this study had a full-term birth (92.6%).

In the unadjusted analysis (data not shown), we observed a significant relationship between ICE and gestational age effect regardless of whether EOD was high (β = 1.19, 95% CI: (0.33, 2.05); p-value = 0.006) or low (β = 0.36, 95% CI: (0.17, 0.55); p-value < 0.001). We note that the strength of this association did not differ significantly between the EOD high and low groups at the 0.05 significance level (p = 0.06).

Controlling for the confounding variables, we observed a significant individual and structural racism interaction effect on gestational age (p = 0.03) (Table 2). In the subgroup analyses, our data showed that, among those with high EOD scores, women who were from households concentrated in the more privileged group had significantly longer gestations (β = 1.07, 95% CI: (0.24, 1.90); p-value = 0.012). However, among those with low EOD scores, there was no significant relationship between ICE and gestational age (β = 0.13, 95% CI: (-0.07, 0.33); p-value = 0.204) (Table 3).

Sensitivity analyses were performed to assess the robustness of the main findings. The EOD by ICE interaction effect remained significant when a three-level categorized EOD was used in the model (p-value = 0.019).

## Discussion

In this analysis, we found that individual experiences of racial discrimination and income and racial polarization measured by the ICE index were significantly associated with gestational age at birth in a sample of nulliparous women in the U.S. We also found that EOD moderated the association between racialized residential segregation and gestational age at birth. Our findings are in line with previous studies examining associations between individual- and structural-level racism separately and adverse birth outcomes.[1,10,23] We extend previous work by examining the association between individual and structural racism together with gestational age, and by extending analyses to include Hispanic women.

In comparison to pregnant women living in the higher income concentration of households, those who lived in households concentrated in deprivation had infants born at significantly earlier gestational ages. We found that about 17% of NH Black women and 10% of Hispanic women in nuMoM2b had ever experienced racial discrimination in at least three social situations, and that higher EOD scores were significantly associated with shorter gestational age at birth for NH Black women only. The significant association between structural level racism and gestational age became insignificant after accounting for maternal race, which indicated the magnitude of associations between this racism measure and gestational age did not differ by race. This reveals the detrimental effects structural racism could have on all races. However, NH Black and Hispanic women were more likely to experience interpersonal discrimination or be treated unfairly, and were more likely to birth infants at earlier gestational ages compared to NH White women.

## Public Health Implications

There is a paucity of studies examining the effects of both structural and individual racism on health outcomes and the experiences of racism and discrimination and adverse pregnancy outcomes among Hispanic women. One qualitative study of Puerto Rican women in Connecticut (n = 29) examined stressors including racial and ethnic discrimination, finding that poverty, food insecurity, low quality education and unsafe environments contributed to poor maternal and child health conditions.[24] Another study compared Black (n = 1,154) and Latina (n = 578) women from the Community Child Health Research Network Study, and found that Latina women who experienced everyday racism a few times a month (medium exposure category) had a higher risk of PTB (adjusted hazard ratio (aHR) = 4.1 (95% CI 1.1–15.5), but no significant relationship was observed among Black women in that study (aHR = 1.5, 95% CI 0.8–2.9).[25] Others reported a significant relationship between experiences of discrimination and low birthweight among Latina (n = 262) and Black (n = 158) young women in New York City, however, Latina women were not examined separately.[26]

We also found no significant differences in gestational age at birth for Hispanic women compared to NH White women despite their socioeconomic disadvantage which could be a demonstration of the "Hispanic paradox", which refers to the seemingly contradictory finding that, despite facing socioeconomic disadvantages, Hispanic individuals in the U.S. tend to have better health outcomes than NH Whites.[27] A large proportion of Hispanic women in this study were from Mexico, but also many participated had other countries of origin. It is possible that the protective effects cited in previous work supporting the Hispanic paradox in Mexicans may have been offset by combining Mexican women in nuMoM2b with women from other countries. Previous work suggests that the Hispanic paradox may not be a universal phenomenon across all Hispanic subgroups, with worse birth outcomes reported in women with Dominican or Puerto Rican heritage.[28] However, it is difficult to interpret our study findings in the context of the Hispanic paradox as Hispanic women had birth outcomes that were comparable to NH White women. This could also be explained by the almost even distribution of women in the second generation in the U.S., or due to more social support received by Hispanic women living in multi-generational households.[29,30]

Shorter gestation is one of the most important predictors of infant health and mortality.[31] Our findings convey the complex role of individual and structural racism on inequalities in birth outcomes. Individual experiences with discrimination and increased levels of stress associated with these experiences have also been shown to have detrimental effects on birth outcomes.[8] The income and racialized residential segregation captured by the ICE measure demonstrated injustices within communities through systematic racism and discrimination which have been shown to contribute to persistent health inequities and disparities that continue to impact communities of minoritized women today.[1,3,10] Thus, examination of these measures is important as they are tied to potential interventions to resolve the health impacts of social inequalities and health disparities. More work is required to further examine the multidimensional nature of racism and its effects on gestational age and birth outcomes. Future research should also focus on experiences of racism and discrimination among Hispanic women, by geographic area of origin, and include measures of acculturation and other factors that may contribute to poor outcomes.

This study builds upon previous findings on racism and pregnancy outcomes by examining the impact of structural racism and individual experience of racism while accounting for individual-level characteristics. Our study also had limitations. We conducted a cross-sectional study, limiting our ability to account for changes in exposure to racism and segregation patterns over time. However, the results from this study offer a glimpse into the impact of racism and discrimination experiences on gestational age at birth. Future studies should examine the effects of exposure to structural racism and individual experiences of racism across the lifetime using longitudinal datasets. Such datasets will be available soon as nuMoM2b is continuing to follow the cohort as part of the National Heart, Lung, and Blood Institutes Heart Health Study (HHS). Secondly, it is important to note that the low percentage of NH Black and Hispanic women in the study may limit the generalizability of the findings. In addition, our study is limited by incomplete data on the length of time the women resided in their neighborhoods, which is an important factor to consider since the impact of exposure to unfavorable socioeconomic conditions throughout the lifetime and even across generations may lead to racial disparities in preterm birth and other adverse birth outcomes.[32–35] Finally, the primiparous women in nuMoM2b were healthy, low-risk, and had the means and agreed to participate in an intensive, longitudinal study. These characteristics may not be representative of the larger populations, and underscore the need for more longitudinal studies in pregnancy that focus enrollment on NH Black and Hispanic women, with considerable heterogeneity within their own racial and ethnic groups to more accurately study — and identify — reasons for disparities in adverse birth outcomes.

We have shown that individual experiences of racial discrimination and income and racial polarization are significantly associated with gestational age at birth in a sample of nulliparous women in the U.S. Further research is needed, including improved conceptualization of multilevel racism in studies examining racial and ethnic disparities in adverse birth outcomes. These studies should include a greater representation of heterogenous NH Black and Hispanic women to better understand this complex relationship.

## Declarations

**CREDIT:** V. Barcelona conceived and supervised the analysis and led the writing. L. Chen and Y. Zhao conducted the statistical analyses. G. Samari, C. Monk, R. McNeil, A. Baccarelli, and R. Wapner contributed to revision and editing of the manuscript. R. McNeil contributed to analysis. R. Wapner was a PI of the original study.

**Funding**: This work was funded by Columbia Population Research Center (CPRC) Seed Pilot Funds Program, Grant # P2CHD058486, from the National Institute of Child Health and Human Development (NICHD). This study was also supported by grant funding from the Eunice Kennedy Shriver National Institute of Child Health and Human Development (NICHD U10 HD063036, U10 HD063072, U10 HD063047, U10 HD063037, U10 HD063041, U10 HD063020, U10 HD063046, U10 HD063048, and U10 HD063053) and the National Heart, Lung, and Blood Institute (U01 HL145358) with supplemental support from the Office of Behavioral and Social Sciences Research. In addition, support was provided by Clinical and Translational Science Institutes: UL1TR001108 and UL1TR000153. The funding sources were not involved in the conduct of this research study or in the preparation of the article.

## Tables

Table 1. Participant characteristics for non-Hispanic Black, non-Hispanic White, and Hispanic women in the Nulliparous Pregnancy Outcomes Study: Mothers-to-Be (nuMoM2b) Study, 2010 – 2013 (n=7,732).

| | Total | Non-Hispanic White | Non-Hispanic Black | Hispanic |
|---|---|---|---|---|
| | n (%) [a] | n (%) | n (%) | n (%) |
| | 7,732 (100) | 5,234 (67.7) | 1,073 (13.9) | 1,425 (18.4) |
| Maternal Age | | | | |
| 10 – 19 | 789 (10.2) | 258 (4.9) | 282 (26.3) | 249 (17.5) |
| 20 – 29 | 4,302 (55.6) | 2,784 (53.2) | 637 (59.4) | 881 (61.8) |
| 30 – 39 | 2,538 (32.8) | 2,114 (40.4) | 142 (13.2) | 282 (19.8) |
| 40 – 49 | 103 (1.3) | 78 (1.5) | 12 (1.1) | 13 (0.9) |
| Missing | 0 (0.0) | 0 (0.0) | 0 (0.0) | 0 (0.0) |
| | | | | |
| Mean (s.d.) | 26.9 (5.6) | 28.2 (5.1) | 23.4 (5.4) | 24.8 (5.5) |
| | | | | |
| Education | | | | |
| Less than HS grad | 575 (7.4) | 200 (3.8) | 185 (17.2) | 190 (13.3) |
| HS grad or GED | 925 (12.0) | 387 (7.4) | 274 (25.5) | 264 (18.5) |
| Some college | 1,531 (19.8) | 782 (14.9) | 320 (29.8) | 429 (30.1) |
| Assoc/Tech degree | 804 (10.4) | 536(10.2) | 111 (10.3) | 157 (11.0) |
| Completed college | 2,156 (27.9) | 1,806 (34.5) | 111 (10.3) | 239 (16.8) |
| Degree work beyond college | 1,736 (22.5) | 1,523 (29.1) | 72 (6.7) | 141 (9.9) |
| Missing | 5 (0.06) | 0 (0.0) | 0 (0.0) | 5 (0.4) |
| | | | | |

| | | | | |
|---|---|---|---|---|
| Smoked tobacco in the three months prior to pregnancy | | | | |
| No | 6,365 (82.3) | 4,353 (83.2) | 788 (73.4) | 1,224 (85.9) |
| Yes | 1,362 (17.6) | 879 (16.8) | 285 (26.6) | 198 (13.9) |
| Missing | 5 (0.06) | 2 (0.04) | 0 (0.0) | 3 (0.2) |
| | | | | |
| Gestational Age (weeks) at delivery, mean (s.d.) | 38.8 (2.1) | 38.9 (1.97) | 38.4 (2.6) | 38.8 (2.0) |
| | | | | |
| Preterm birth (<37 weeks) | | | | |
| No | 7092 (91.7) | 4,829 (92.3) | 944 (88.0) | 1,319 (92.6) |
| Yes | 640 (8.3) | 405 (7.7) | 129 (12.0) | 106 (7.4) |
| Missing | 0 (0.0) | 0 (0.0) | 0 (0.0) | 0 (0.0) |
| | | | | |
| Site | | | | |
| Case Western Reserve University | 295 (3.8) | 127 (2.4) | 138 (12.9) | 30 (2.1) |
| Columbia University | 1,438 (18.6) | 760 (14.5) | 122 (11.4) | 556 (39.0) |
| Indiana University | 653 (8.5) | 386 (7.4) | 198 (18.5) | 69 (4.8) |
| Magee-Women's Hospital | 1,130 (14.6) | 812 (15.5) | 266 (24.8) | 52 (3.6) |
| Northwestern University | 1,095 (14.2) | 946 (18.1) | 50 (4.7) | 99 (6.9) |
| University of California, Irvine | 595 (7.7) | 210 (4.0) | 54 (5.0) | 331 (23.2) |
| University of Pennsylvania | 709 (9.2) | 408 (7.8) | 237 (22.1) | 64 (4.5) |
| University of Utah | 1,817 (23.5) | 1,585 (30.3) | 8 (0.7) | 224 (15.7) |
| Missing | 0 (0.0) | 0 (0.0) | 0 (0.0) | 0 (0.0) |

| | | | | |
|---|---|---|---|---|
| | | | | |
| Experiences of Discrimination (EOD)<br><br>(Range: (0,9)] | | | | |
| Low (EOD < 3) | 7,305 (91.0) | 5,027 (96.0) | 827 (77.1) | 1,181 (82.9) |
| High (EOD | 426 (5.5) | 104 (2.0) | 187 (17.4) | 135 (9.5) |
| Missing | 271 (3.5) | 103 (2.0) | 59 (5.5) | 109 (7.6) |
| | | | | |
| Mean (s.d.) | 0.4 (1.0) | 0.2 (0.7) | 1.1 (1.7) | 0.7 (1.3) |
| | | | | |
| Index of Concentration at the Extremes (ICE) (Range: [-1,1]) | | | | |
| Deprivation (ICE ) | 1,856 (24.0) | 481 (9.2) | 816 (76.0) | 559 (39.2) |
| Privilege (ICE ) | 5,871 (75.9) | 4,752 (90.8) | 256 (23.9) | 863 (60.6) |
| Missing | 5 (0.06) | 1 (0.02) | 1 (0.1) | 3 (0.2) |
| | | | | |
| Mean (s.d.) | 0.1 (0.3) | 0.2 (0.2) | -0.2 (0.3) | 0.02 (0.3) |

[a] Numbers may not sum to 100 due to rounding

[b] s.d.=standard deviation

**Table 2: Individual experiences of discrimination moderate the effect of structural racism on gestational age at birth.**

| Predictors | Estimate (β) [a] | 95% CI [b] | p |
|---|---|---|---|
| (Intercept) | 39.32 | (39.02, 39.61) | **<0.001** |
| ICE | 0.14 | (-0.06, 0.34) | 0.175 |
| EOD High | -0.07 | (-0.27, 0.12) | 0.467 |
| Maternal Age | -0.03 | (-0.04, -0.02) | **<0.001** |
| Education | | | |
| HS Grad or GED | 0.08 | (-0.13, 0.30) | 0.451 |
| Some College | 0.26 | (0.05, 0.46) | **0.014** |
| Associate/Tech Degree | 0.34 | (0.10, 0.57) | **0.005** |
| Completed College | 0.57 | (0.35, 0.79) | **<0.001** |
| Degree beyond College | 0.56 | (0.32, 0.81) | **<0.001** |
| Smoking | -0.02 | (-0.14, 0.11) | 0.793 |
| Gestational Hypertension | -0.23 | (-0.39, -0.08) | **0.003** |
| Gestational Preeclampsia | -1.72 | (-1.95, -1.49) | **<0.001** |
| Gestational Diabetes | -0.55 | (-0.78, -0.31) | **<0.001** |
| ICE * EOD High | 0.75 | (0.08, 1.43) | **0.029** |

[a] β = beta, [b] CI = Confidence Interval; the CI was approximated using the Wald method.

**Table 3. Individual experiences of discrimination moderate the effect of structural racism on gestational age.**

| | Low EOD (n=7005) | | | High EOD (n=423) | | |
|---|---|---|---|---|---|---|
| **Predictors** | **Estimates** | **95% CI** | **p** | **Estimates** | **95% CI** | **p** |
| (Intercept) | 39.20 | (38.90 – 39.50) | **<0.001** | 40.46 | (39.17 – 41.75) | **<0.001** |
| ICE | 0.13 | (-0.07 – 0.33) | 0.204 | 1.07 | (0.24 – 1.90) | **0.012** |
| Maternal Age | -0.02 | (-0.03 – -0.01) | **<0.001** | -0.07 | (-0.12 – -0.02) | **0.007** |
| Education | | | | | | |
| HS Grad or GED | 0.13 | (-0.09 – 0.35) | 0.235 | -0.64 | (-1.67 – 0.40) | 0.226 |
| Some College | 0.28 | (0.07 – 0.49) | **0.009** | -0.05 | (-1.01 – 0.92) | 0.924 |
| Associate/Tech Degree | 0.36 | (0.12 – 0.60) | **0.003** | 0.00 | (-1.10 – 1.10) | 1.000 |
| Completed College | 0.57 | (0.34 – 0.80) | **<0.001** | 0.70 | (-0.39 – 1.79) | 0.207 |
| Degree beyond College | 0.57 | (0.33 – 0.82) | **<0.001** | 0.35 | (-0.82 – 1.52) | 0.561 |
| Smoking | -0.04 | (-0.17 – 0.09) | 0.551 | 0.18 | (-0.36 – 0.72) | 0.522 |
| Gestational Hypertension | -0.23 | (-0.39 – -0.07) | **0.004** | -0.37 | (-1.16 – 0.43) | 0.368 |
| Gestational Preeclampsia | -1.76 | (-1.99 – -1.52) | **<0.001** | -1.14 | (-2.24 – -0.03) | **0.043** |
| Gestational Diabetes | -0.60 | (-0.84 – -0.36) | **<0.001** | 0.10 | (-0.88 – 1.07) | 0.847 |

## Figures

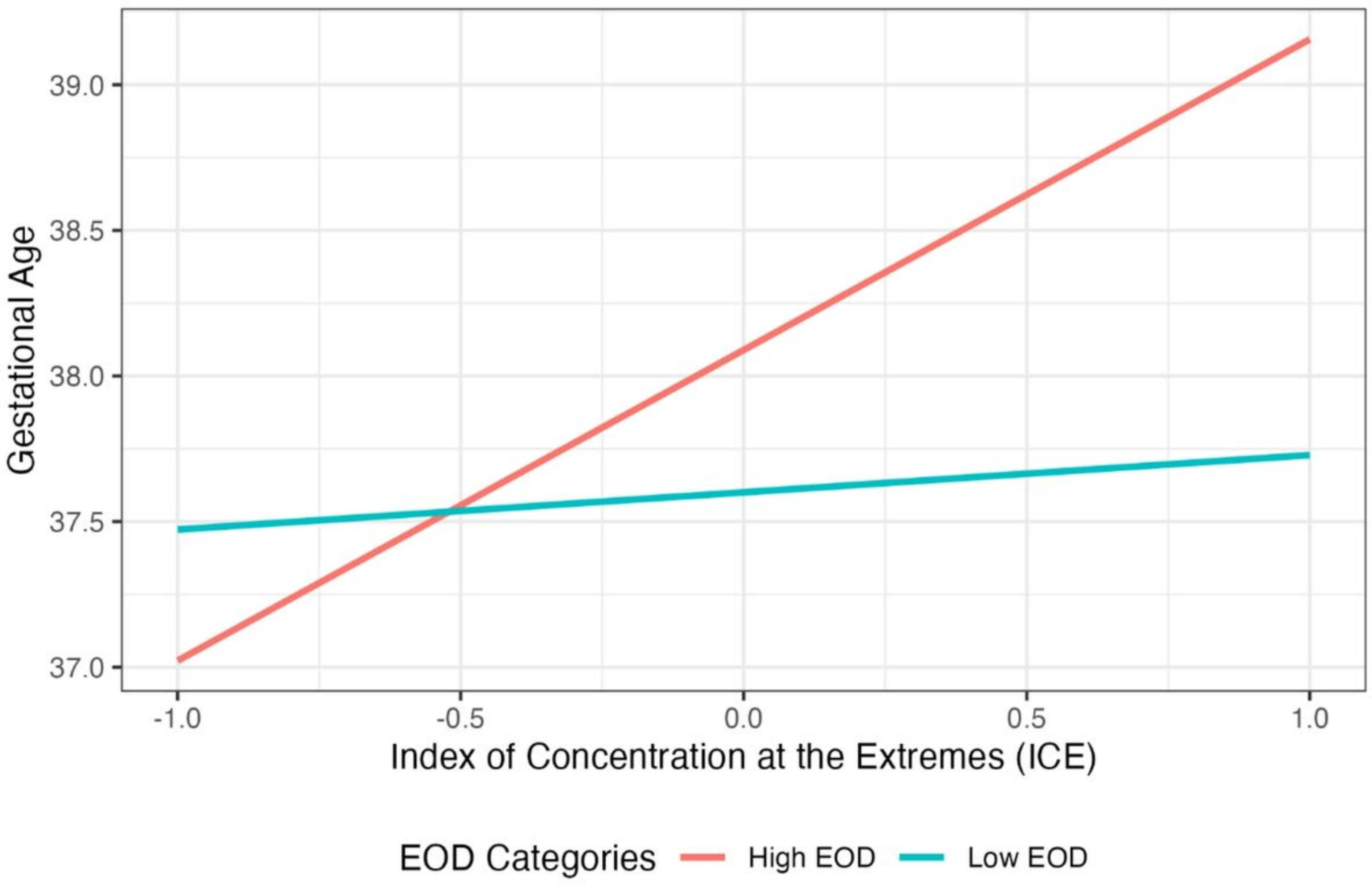

**Figure 1**

Individual experiences of discrimination moderate the association between structural racism and gestational age at birth. A significant relationship between ICE and gestational age was found with high EOD (p-value=0.012), but not with low EOD (p-value=0.204).
